# Cost-Effectiveness and Patient Outcomes of Injectable Collagenase to Treat Dupuytren’s Contracture

**DOI:** 10.7759/cureus.20530

**Published:** 2021-12-20

**Authors:** Rajpreet S Sahemey, Govind S Dhillon, Karanjeet S Sagoo, Kuntrapaka Srinivas

**Affiliations:** 1 Trauma & Orthopaedics, University Hospital Coventry, Coventry, GBR; 2 Orthopaedic Surgery, Lister Hospital, Hertfordshire, GBR; 3 Surgery, Bedford Hospital, Bedford, GBR; 4 Orthopaedics, University Hospitals Birmingham, Birmingham, GBR

**Keywords:** contracture release, fasciectomy, cost assessment, dupuytren’s disease, collagenase

## Abstract

Introduction

Dupuytren’s contracture is a disabling and progressive flexion contracture of the hand that is often treated by a surgical release. Collagenase clostridium histolyticum injection (CCH-I) was introduced to the UK in 2011 as an alternative and less invasive treatment for contracture. The purpose of this study was to evaluate the cost-effectiveness and patient-reported outcome measures (PROMs) of treating Dupuytren’s contracture with collagenase compared to surgery.

Methods

A retrospective review identified 151 patients who underwent CCH-I (n=94), limited fasciectomy (LF; n=38) and percutaneous needle fasciotomy (PNF; n=19). Outcomes included PROMs (satisfaction, QuickDASH), complication rates (recurrence, reintervention) and direct costs.

Results

Standardised treatment costs for CCH-I, LF and PNF were £1,125.82, £3,438.28 and £1,143.32 respectively. Collagenase presented a cost-benefit of £88,205 had the LF/PNF group undergone CCH-I. At a mean six-year follow-up, there were no significant differences in complication rates (=0.621) or QuickDASH scores (p=0.157). Collagenase-treated patients reported the highest satisfaction and lowest recurrence rates.

Discussion

Collagenase presents a significant cost reduction with superior PROMs relative to surgery for treating single-digit contracture.

Conclusion

Outpatient CCH-I is a cost-effective treatment with fewer clinical encounters, a similar risk profile to LF/PNF and high levels of patient satisfaction, which warrants serious consideration in light of overburdened waiting lists due to COVID-19.

## Introduction

Dupuytren’s contracture (DC) of the hand is a disabling and incurable disorder causing progressive flexion contracture of the digits, impairing hand function and diminishing the quality of life [1]. Treatment focuses on deformity correction to improve function, which can be achieved by excision of the thickened cords. Limited fasciectomy (LF) and its variations remain the most widely accepted standard of care and are performed in up to 90% of patients [2]. While open surgery is effective in managing contracture, these procedures may necessitate skin graft and occasionally run the risk of neurovascular injury, prolonged wound healing and extensive hand therapy [3]. Fasciectomy involves lengthy operating times, often performed under general or regional anaesthesia, and has a 30%-50% risk of recurrence [4]. Less invasive treatments including percutaneous needle fasciotomy (PNF) are currently offered as part of the treatment algorithm in the UK [5]. This procedure involves the blind division of cords using a hypodermic needle though carries the risk of neurovascular injury [6].

Collagenase Clostridium histolyticum injection (CCH-I) was introduced in 2011 as an enzymatic treatment option for DC. The drug is injected into the affected cords causing them to soften, allowing for rupture by manipulation of the digit, with or without local anaesthesia. The entire treatment delivery can be performed on an outpatient basis without the need for operating theatre capability [7]. Compared with surgery, CCH-I is effective, well tolerated and has lower complication and recurrence rates at three years [8].

Despite the advantage of CCH-I, there has been reluctance towards its use across Europe and has been withdrawn from the market since March 2020 for commercial and clinical reasons despite ongoing use in the USA. Concerns were driven by the long-term efficacy of this relatively new treatment and also due to the high one-off price of the drug, which is not funded by a payment-by-results system incorporated in many healthcare systems such as the National Health Service (NHS) [9,10]. The aim of this study was to compare treatment costs, patient-reported outcome measures (PROMs) and recurrence of DC at a minimum of 2.5 years post-treatment with CCH-I versus surgery.

## Materials and methods

Patients and settings

We performed a retrospective review of all patients treated for DC at our tertiary hand surgery department (three consultant surgeons) in a teaching hospital between January 2014 and December 2018. Patients were eligible if they were undergoing unilateral CCH-I, LF or PNF as the primary treatment of a symptomatic single-digit contracture with a positive Hueston's tabletop test (Tubiana grading was not formally assessed). Patients were excluded if their encounter was for a revision procedure, had undergone any of the treatments previously or treatment of multiple digits. All patients included in the final analysis had completed the treatment course for their presentation and were discharged from hand therapy. The minimum follow-up period was two years, which has been defined as a threshold for early recurrence [11]. Data collected from medical records included demographics, number of surgeon and hand therapy-led outpatient visits, complications and re-operations. Institutional Review Board (IRB) approval was not required in accordance with National Research Ethics Service (United Kingdom) guidance on the use of anonymised data collected retrospectively as part of routine clinical care.

Cost analysis

Standardised costs of procedures, outpatient appointments, splinting and therapy were provided by the hospital finance department; adapted from the NHS National Tariff (2016/17). The HRG (Health Resource Group) reference cost codes for undergoing a day case LF procedure were HB53Z (£2,419) and HB51Z (£4,743). At our unit, approximately 75% fasciectomies are booked under the HB53Z code and 25% under HB51Z, therefore in our model an average cost of £3,000 was used. Similarly, 95% of PNF day case procedures are coded HB55B (£726) and the remainder HB55C (£816); thus an average cost of £811.50 was applied. A single vial of collagenase costs £572 and is booked as a day case injection (reference code HB56C; £222). The cost of the initial consultation was £129 (reference code WF01B) and £77 for each surgeon-led appointment (reference code 110). Each hand therapy episode cost £38.82 with an additional £10 estimated for splint provision [9].

Outcome measures

The primary outcome measure was a cost analysis of treatments. Secondary measures included PROMs, recurrence (contracture >20° at any point during follow-up), reoperation and complications. Complications were categorised as ‘major’ if they required intervention (skin tear, injury to tendon, nerve or artery, complex regional pain syndrome [CRPS], or wound complication). Minor complications included oedema, pain and paraesthesia. All PROMs were collected via telephone and consisted of:

· Quick disabilities of the arm, shoulder and hand (QuickDASH) score

· Overall satisfaction (Yes/No)

· Willingness to repeat treatment (Yes/No)

Treatments

**Figure 1:**
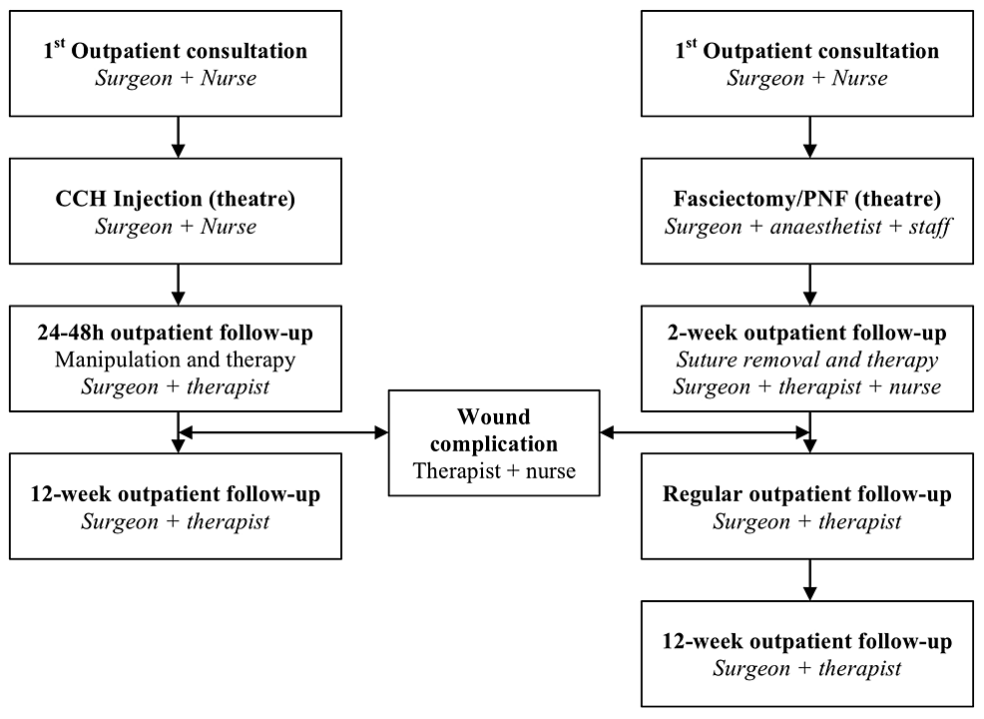
Treatment algorithm showing the sequential treatment stages for Dupuytrens contracture with either collagenase Clostridium histolyticum (CCH) injection, or surgically with fasciectomy or percutaneous needle fasciotomy (PNF)

Patients attend an initial surgeon-led consultation where a decision to treat is made and the patient is placed onto a waiting list (Figure 1).

At our centre CCH-I is administered in a vacant theatre for logistical reasons due to the availability of an emergency trolley and refrigeration of drugs. Typically a surgeon, nurse and healthcare assistant are present. As a result, the administration cost for CCH-I is uniformly higher than if it were administered in an outpatient setting. The procedure is booked as a 15-minute injection allowing multiple patients to be treated per session. The patient undergoes CCH-I (Xiapex®, SOBI, Stockholm, Sweden) according to the manufacturer’s instruction without local anaesthesia to the metacarpophalangeal joint (MCPJ) or proximal interphalangeal joint (PIPJ) contracture. The patient visits the outpatient department within 48 hours where the surgeon performs definitive manipulation under local anaesthetic. A hand therapist commences rehabilitation in the same appointment and provides a thermoplastic night splint in extension to be used for eight weeks. If skin tear is encountered during manipulation, oral antibiotics are provided and follow-up with the therapist is necessitated as indicated. A final consult is carried out at 16 weeks.

Patients on the LF/PNF surgical pathway are booked as a 90-minute day case procedure under a locoregional or general anaesthesia and using an arm tourniquet at 250 mmHg. For LF, a Brunner skin incision is used which is closed with interrupted nylon 4-0 sutures and the hand is immobilised in a short-arm extension slab. At two weeks, the slab and sutures are removed and the therapy commences. In the case of PNF, a 25-gauge needle is used to perforate the cord until clinical rupture is achieved. Fasciotomy is performed in the theatre complex due to the availability of procedural space, drugs cabinet (for local anaesthetic storage) and emergency trolley as per local guidelines. Postoperatively for both groups, the patient works on scar management, oedema control and range of motion exercises under therapist supervision. Similarly, a night splint is provided for eight weeks and wound issues are closely monitored. Subsequent visits vary depending on progression.

Statistical analysis

Sample size calculation (using Shapiro Wilk test for normality) recommended 132 patients consisting of 44 surgically treated and 88 CCH-I treated patients to provide 90% power (α=0.05; β=0.1) to detect a 10% difference in QuickDASH scores between groups. Comparisons of mean QuickDASH scores and complication rates were made using Student’s t-test. The level of significance was set at p<0.05. Data analysis was performed using SPSS (Statistical Package for the Social Sciences, version 22.0, SPSS Inc., Chicago, IL, USA).

## Results

This study comprised a total of 151 patients (104 male; 47 female) who were treated for symptomatic single-digit DC at our unit. Of these, 94 were treated with CCH-I, 38 with LF and 19 with PNF. The Median follow-up was 6.3 years (interquartile range, 5.8-6.8 years). Treatment group characteristics and outcomes are highlighted in Table 1. The mean overall QuickDASH scores were similar at final follow-up for both the CCH-I and LF/PNF groups. At a mean of six years follow-up, patients reported comparably low levels of impairment and disability (CCH-I, 8.8 ± 13.2 versus LF/PNF, 11.6 ± 10; p=0.157). There were no significant differences in complication rates (p=0.621). Within each treatment group, there were no early returns to the theatre or overnight admission. Post-treatment contracture measurement data were not specifically collected in this study unless the patient presented to outpatient follow-up with recurrence, at which point contracture measurements were recorded.

**Table 1:** Treatment group summaries and outcomes ^a^American Society of Anesthesiologists physical status classification; ^b^Disabilities of the arm, shoulder and hand Score; ^c^Collagenase Clostridium Histolyticum injection; ^d^Limited fasciectomy/percutaneous needle fasciotomy

Treatment	N	M:F	Mean age	ASA^a^ score	Mean cost (£GBP)	Recurrence (>20°)	Reoperation	Complication	Mean final QuickDASH^b^	Satisfaction	Would repeat
CCH-I^c^	94	75:19	65.1	2.4	1,125.82	8 (8.5%)	10 (10.6%)	35 (37.2%)	8.8	87.2%	87.2%
LF/PNF^d^	57	29:28	65.6	1.5	2,673.29	6 (10.5%)	6 (10.5%)	19 (33.3%)	11.6	82.5%	84.2%

Cost comparison

**Figure 2:**
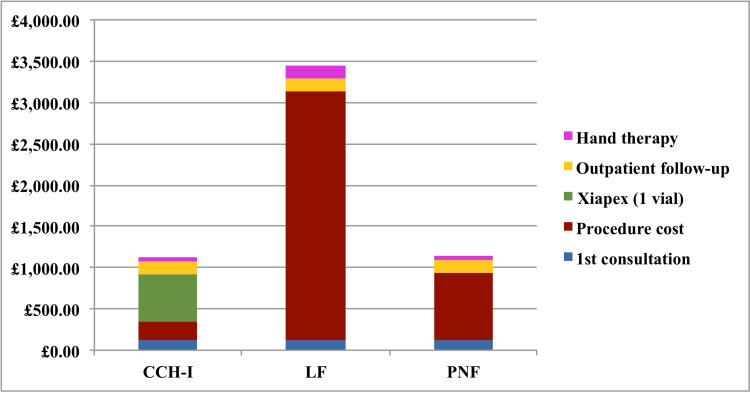
Cumulative costs of treatment Bar chart illustrating the breakdown of total costs per treatment for collagenase Clostridium histolyticum (CCH); limited fasciectomy (LF) and percutaneous needle fasciotomy (PNF)

Excluding complications, the per-patient average total treatment costs from initial consultation to final follow-up were £1,125.82 for CCH-I and £2,673.29 for LF/PNF (LF, 3,438.28; PNF, £1,143.32). Figure 2 demonstrates the cumulative costs per treatment. The treatment cost for the LF/PNF group was £152,377. Had these 57 patients been treated with CCH-I then the estimated treatment cost would be £64,172. This suggests a potential cost saving of £88,205 with CCH-I treatment, or a £1,547 cost saving per patient. Overall, CCH-I had significantly fewer outpatient follow-up episodes (mean, 2.1) compared with the LF/PNF group (mean 3.4, range 2-8). Including a 37% risk of complication and cost of re-intervention with an LF procedure, we estimate that the additional burden for CCH-I increases by £423 per patient. Therefore with the projected treatment and complication costs, there is still a £64,141 cost-benefit of using CCH-I.

Collagenase

The CCH-I group consisted of 94 patients (75 male; 19 female) with a mean age of 65.1 ± 11.3 years and a mean ASA grade of 2.4 ± 1. This group had a greater complication rate (37.2%) compared with the surgical cohort (summarised in Table 2). Skin tear of the digit during outpatient manipulation occurred in 34 patients. All wounds healed with conservative management by four weeks though these patients required frequent monitoring by therapist or nurse-led clinics until fully healed. These additional appointments are accounted for (mean 2.1 additional appointments). Three of these patients developed severe pain at the wound site, which subsided after four months. A further patient developed CRPS requiring oral medication and hand therapy, which resolved after eight months. The rate of >20°contracture recurrence was lower (8.5%) compared with the LF/PNF group. Ten patients (10.6%) underwent reoperation with LF and included eight patients with a >20° contracture and two patients with a 10° contracture recurrence. The mean time to re-operation was 2.3 years (interquartile range, 1.1-4 years). All patients would have been suitable for repeat treatment with CCH-I though they were “not willing” to repeat treatment with CCH-I if offered. Reasons for this included an unwillingness to attend multiple appointments for injection and manipulation. Patient satisfaction was greater (87.2%) than the LF/PNF group (82.5%).

**Table 2:** Complications by treatment type ^a^Collagenase Clostridium histolyticum injection; ^b^Limited fasciectomy/percutaneous needle fasciotomy; ^c^Complex regional pain syndrome

Complication	CCH-I^a^ n (%)	LF/PNF^b^ n (%)
Skin tear	34 (36)	
Wound issue		8 (14)
Pain	3 (3)	2 (4)
CRPS^c^	1 (1)	3 (5)
Paraesthesia		3 (5)
Hypersensitivity		2 (4)
Swelling		1 (2)

Surgical group

The LF/PNF group included 57 patients (29 male; 28 female) with a mean age of 65.6 ± 12.4 years. Mean ASA grade was slightly lower at 1.5 ± 0.6. Despite a lower satisfaction rate, there were fewer complications (33.3%). Eight patients (14%) developed superficial wound infections following LF, which were treated with oral antibiotics and more frequent outpatient reviews until satisfactory. Three patients developed CRPS, which resolved after 12 months. Five patients developed paraesthesia and hypersensitivity; three of these patients continued to have persistent altered sensation around the surgical wound. A further patient had digital swelling that settled by three months. We encountered similar reoperation rates (10.5%), which were due to >20° contracture recurrence at a mean time of 18.5 ± 7.5 months. Four of these recurrences were from the PNF subgroup (21% recurrence) and two from the LF subgroup (5% recurrence). The overall recurrence rate with LF/PNF was 10.5%. With the exception of one repeat PNF, five revision fasciectomies were performed. Though patient satisfaction was lowest for the PNF subgroup (79%), most were willing to repeat treatment if necessary (94.7%).

## Discussion

Treatment of single-digit DC with CCH-I represents a significant cost reduction relative to surgery and is a similar finding to other studies that also demonstrated cost reductions of up to 70% [9,12,13]. Our study demonstrates significantly better PROMs for CCH-I compared with surgical alternatives from at least 2.5 years following treatment. The total cost of treatment for a single patient remains lower than LF if two vials of the enzyme are needed (£1,740.72) though becomes a more expensive option compared with PNF (£1,356). None of the CCH-I patients required more than one vial of collagenase to achieve satisfactory contracture correction. This is supported by similar experiences from others reporting that 1-1.4 injections are sufficient to achieve a correction [9,14]. Murphy et al. [15] suggested that injection into multiple areas may yield better results though the results from our collagenase cohort reflect the single-site injection technique recommended by the manufacturer.

The greatest costs of surgery arise from the procedure itself with LF costing £2,419, representing theatre facility costs, professional fees (surgeon, anaesthesia, nursing) and day-case hospital charges. This study did not collect operating time data however the average duration of an LF has been estimated to be approximately 60 minutes, meaning that four to five fasciectomies can be performed per session. In contrast, up to 30 patients can be potentially treated with CCH-I per session, or up to 20 patients per outpatient clinic [12]. Patients can also be treated for bilateral disease in the same sitting, which is a substantial benefit over surgery. A further advantage of CCH-I is the greater applicability of DC treatment for those who would have otherwise been unsuitable for anaesthesia, i.e. high ASA grade or significant comorbidities. This was reflected in our demographic with the CCH-I group having a higher mean ASA grade.

The greatest individual recurrence rates were encountered in the PNF group (21%) and the lowest for LF-treated patients (5%). Chen [16] found that PNF had the highest recurrence rate of 60% when compared with LF and CCH-I. Even though it is a relatively cheaper treatment, high recurrence rates at five years may make it an unacceptable option for both patients and surgeons [17]. Dutta [18] reviewed DC treatment options and concluded that due to the percutaneous nature of the procedure, neurovascular bundles are highly susceptible to injury and should be avoided in PIPJ contracture or severe deformity because of the proximity of digital nerves to the cord. The accepted recurrence rate of collagenase remains unclear with figures ranging from 10% to 47% reported in the literature [8,16]. Our recurrence rate of 10.6% remains in keeping with established results.

The rate of significant complications with CCH-I is reportedly rare [19]. The commonest complication across both treatment groups were skin issues, namely skin tears (36%) in the CCH-I group, and delayed wound healing (14%) due to dehiscence or infection in the LF/PNF group. Skin tear following CCH-I manipulation is a common complication, particularly in PIPJ contracture or a contracture >70° and has a reported rate of up to 19% [20]. In our study, this complication accounted for an additional 2.5 outpatient visits for wound management and dressings. An explanation for our higher rate of skin tears could be due to patients having more severe contractures and patients opting for a less invasive treatment option. Thus, this complication can be predicted in those with high-risk contractures and such patients may be counselled about treatment options accordingly.

There remains a lack of consensus on the postoperative rehabilitation of DC. In the present study, all DC patients adhered to a night-time splinting protocol for eight weeks. The CCH-I group was encouraged to actively mobilise post-manipulation whereas the surgical cohort was additionally immobilised in a temporary slab for two weeks postoperatively, which may account for their relatively worse QuickDASH and satisfaction scores. Recent trends in rehabilitation, supported by Cochrane review, suggest that postoperative splinting for DC is falling out of favour and may impair outcomes by reducing early active flexion [18,21]. Kemler [22] found that finger flexion was worse in a splinted group of patients than with patients undergoing hand therapy alone and further hypothesised that scar tissue formed in extension may limit movement whereas scar tissue produced in a mobile digit is laid down in a more functional manner to allow flexion. The evidence, however, is of limited quality and lacks prolonged follow-up periods. Our protocol of night splinting for eight weeks was based on studies, which suggest that the contracture resolution is dependent on total orthosis dosage [23]. Furthermore, some suggest that night-time splinting after DC correction does not significantly worsen DASH scores or patient satisfaction at one year [24]. Overall the variability in the number of outpatient follow-up appointments by the surgeon and hand therapist, particularly in the LF/PNF group, highlights the need to streamline rehabilitation and follow-up pathways. The current coronavirus disease 2019 (COVID-19) pandemic has resulted in an unprecedented reformation of healthcare service provision. Hospital providers have had to restructure outpatient care to comply with social distancing and have stressed the importance of reducing excessive and unnecessary patient encounters and the number of persons in departments at any one time. The cost-effectiveness and benefits of telemedicine, provision of therapy instruction leaflets, and patient-initiated follow-up have been recognised and are being utilised at an exponential rate [25].

Our study has limitations. While the primary outcomes in this study included PROMs, contracture data would be essential to determine the clinical efficacy of interventions. Recurrence and reoperation rates are not synonymous. Some patients may consider their treatment to be a failure due to recurrence however they may wish not to undergo further treatment. In our series all CCH-I patients who required treatment for recurrence (n=10) underwent a fasciectomy. These patients would have been suitable for repeat collagenase injection however between 2015 and 2016, the regional commissioning group suspended funding for collagenase. However, for patients who would not be suitable for surgical re-intervention, it is possible that the treatment cost of CCH-I would exceed that of LF with recurrence. Finally, we did not account for potential cost differences for treating multiple digits in a single sitting. As the cost of fasciectomy remains fixed there are substantial economies of scale to be achieved when treating two or more digits in the same sitting which makes surgical fasciectomy a more cost-effective treatment for the multi-digit disease.

## Conclusions

This is the first study to report long-term patient-reported outcomes and cost-effectiveness of CCH-I compared with surgery at a mean follow-up of six years. The study suggests that CCH-I is cost-effective, associated with better patient outcomes and high satisfaction rates when compared with surgery, even when accounting for complications. Cost benefits arise from a lower treatment administration price and fewer outpatient visits. Collagenase treatment can be delivered from the outpatient setting entirely without the need for hospitalisation. The ability to treat a greater number of ambulatory patients with comorbidities per session with CCH-I can generate additional revenue for healthcare providers and further help to reduce increasingly overburdened waiting lists as a consequence of COVID-19. The potential optimisation of scarce resources and cost efficiency at the present moment represents a significant financial advantage for the NHS. As such, this article recommends steps towards the reapproval of the drug in the UK.
